# Glioma FMISO PET/MR Imaging Concurrent with Antiangiogenic Therapy: Molecular Imaging as a Clinical Tool in the Burgeoning Era of Personalized Medicine

**DOI:** 10.3390/biomedicines4040024

**Published:** 2016-10-31

**Authors:** Ramon F. Barajas, Kenneth A. Krohn, Jeanne M. Link, Randall A. Hawkins, Jennifer L. Clarke, Miguel H. Pampaloni, Soonmee Cha

**Affiliations:** 1 Department of Radiology, Oregon Health & Science University, 3181 SW Sam Jackson Park Rd, Portland, OR 97239, USA; barajas@ohsu.edu (R.F.B.); krohke@ohsu.edu (K.A.K.); lijea@ohsu.edu (J.M.L.); 2 Advanced Imaging Research Center, Oregon Health & Science University, 3181 SW Sam Jackson Park Rd, Portland, OR 97239, USA; 3 Radiochemistry Research Center, Oregon Health & Science University, 3181 SW Sam Jackson Park Rd, Portland, OR 97239, USA; 4 Department of Radiology and Biomedical Imaging, University of California, San Francisco, 505 Parnassus Avenue, M-391, San Francisco, CA 94143-0628, USA; randall.hawkins@ucsf.edu (R.A.H.); miguel.Pampaloni@ucsf.edu (M.H.P.); 5 Neurological Surgery, University of California, San Francisco, 505 Parnassus Ave., Room 779 M, San Francisco, CA 94143-0112, USA; ClarkeJ@neurosurg.ucsf.edu

**Keywords:** glioma, progression, pseudoprogression, FMISO, PET, MRI, bevacizumab, reduced diffusion, ADC

## Abstract

The purpose of this article is to provide a focused overview of the current use of positron emission tomography (PET) molecular imaging in the burgeoning era of personalized medicine in the treatment of patients with glioma. Specifically, we demonstrate the utility of PET imaging as a tool for personalized diagnosis and therapy by highlighting a case series of four patients with recurrent high grade glioma who underwent 18F-fluoromisonidazole (FMISO) PET/MR (magnetic resonance) imaging through the course of antiangiogenic therapy. Three distinct features were observed from this small cohort of patients. First, the presence of pseudoprogression was retrospectively associated with the absence of hypoxia. Second, a subgroup of patients with recurrent high grade glioma undergoing bevacizumab therapy demonstrated disease progression characterized by an enlarging nonenhancing mass with newly developed reduced diffusion, lack of hypoxia, and preserved cerebral blood volume. Finally, a reduction in hypoxic volume was observed concurrent with therapy in all patients with recurrent tumor, and markedly so in two patients that developed a nonenhancing reduced diffusion mass. This case series demonstrates how medical imaging has the potential to influence personalized medicine in several key aspects, especially involving molecular PET imaging for personalized diagnosis, patient specific disease prognosis, and therapeutic monitoring.

## 1. Introduction

The previous decade of biomedical research has fundamentally changed the understanding of human diseases. Among numerous ground breaking scientific advances, the discovery and mapping of the human genome has produced great strides in the development of targeted molecular therapies of numerous disease processes. To this end, the concept of personalized medicine has emerged.

“Personalized medicine” (PM) can be defined as an approach to disease prevention and treatment that takes into account differences in people’s genes, environments, and lifestyles. PM may not necessarily be a new concept, however, its application in the 21st century is producing a paradigm shift in patient care. The purposeful and integrative approach to health care decision making utilizing patient specific, rather than disease specific statistical criteria is a necessarily derived byproduct of our current model for medical practice. The utilization of PM has involved the systematic examination of genomic information of individual patient’s disease process to optimize therapy. A key component of PM is translating the science of medical- and pharmaco-genomics into clinical practice. The successful application of these principles allows for therapeutic targeting of defined sub-populations of disease processes based upon genetics and biomarkers of phenotypes, that may ultimately improve clinical outcomes [1].

As demonstrated by the current success of trastuzumab [2,3,4,5,6,7,8,9,10,11,12,13,14,15], a key aspect of PM is the identification of therapeutically relevant subgroups/phenotypes of a disease processes. The sub-classification of disease is most frequently accomplished through the use of genotyping of individuals and/or relevant tissue specimens. While this is increasingly practical, it is fraught with confounders (tumor heterogeneity, epigenetic factors, and sampling bias; to name a few) when applied in oncology. As such, molecular imaging has tremendous potential as a tool for individualized diagnosis, predicting clinical outcomes, and selecting targeted therapeutic agents for various disease processes.

Over the last few decades molecular imaging has emerged as a tool for investigating in-vivo biological processes. Molecular imaging has evolved from the integration of molecular biology, radiopharmacology, and medical imaging to provide a noninvasive means to visualize cellular function and activity of biological processes. Positron emission tomography (PET) is a widely utilized molecular imaging modality with the potential to directly contribute to PM.

In high grade gliomas the presence of tissue hypoxia is a hallmark of biological aggressiveness. The presence of tissue hypoxia, mediated through expression of hypoxia inducible factor-1 alpha (HIF-1a), stimulates a number of biological processes including vascular endothelial growth factor (VEGF) mediated angiogenesis which has been suggested to drive local recurrence of disease [16,17,18,19]. Additionally, hypoxia limits the efficacy of standard of care chemoradiotherapy. A better understanding of treatment related changes in regional tumor hypoxia may allow for the development of more effective patient-specific therapies. To this end, 18F-fluoromisonidazole (FMISO) PET imaging has been developed as a means of noninvasively quantifying chronic tissue hypoxia [16].

Bevacizumab, a recombinant humanized monoclonal antibody against VEGF, is widely used as an angiogenesis inhibitor in the setting of glioma recurrence. This line of therapy seeks to disrupt and normalize neoplastic vascular formation in an attempt to prevent further tumor growth. Given the interdependent relationship between chronic tissue hypoxia and angiogenesis within recurrent high grade glioma we hypothesized that the pre-bevacizumab quantification of hypoxic tumor burden may be useful in risk stratification.

The purpose of this article is to provide a focused overview of the current use of PET molecular imaging in the burgeoning era of PM in the treatment of patients with glioma. Specifically, we demonstrate the utility of PET imaging as a tool for personalized diagnosis and monitoring of therapy by highlighting a case series of four patients with recurrent high grade glioma who underwent FMISO PET/Magnetic Resonance (MR) imaging through the course of antiangiogenic (bevacizumab) therapy.

## 2. Experimental Section

Four patients with a history of high grade glioma presented with clinical and MR imaging evidence of disease progression (Table 1). All patients had previously undergone maximal safe resection, fractionated external beam radiation therapy, and temozolomide therapy. Upon the diagnosis of glioma recurrence, all patients underwent bevacizumab monotherapy (10 mg/kg IV every two weeks). Following the course of bevacizumab therapy, three of the four patients exhibited clinical and imaging findings that were consistent with tumor recurrence. The fourth patient’s clinical and imaging course was consistent with pseudoprogression. We have previously reported the imaging findings for one patient from the tumor recurrence group [19].

One week prior to initiation and concurrent with bevacizumab therapy all patients underwent Institutional Review Board approved FMISO PET/MR imaging. Following informed written consent, the patients were administered 259 MBq FMISO intravenously. Simultaneous PET/MR imaging was performed on a 3T investigational General Electric (GE) scanner 90 min following FMISO administration. Forty-minute PET emission imaging was performed with time-of-flight reconstruction. Attenuation correction utilized patient-specific T1-weighted map registered with segmented bone from a head CT image template. MR imaging sequences included pre-contrast axial T1 (567/5 millisecond (ms), TR/TE; 5/0 0 Slice Thickness/Skip (mm)), 3D CUBE Fluid-attenuated inversion recovery (FLAIR) (5.34/163/2375 ms, TR/TE/TI; 1/0 mm), 3D T2 (3000/90 ms; 1/0 mm), axial diffusion weighted imaging (DWI) (8000/5 ms; 2/0 mm; 1000 second (s)/mm^2^ B-value), dynamic susceptibility weighted contrast enhanced (DSC) perfusion imaging (1400/25 ms; 3/0 mm; 35° flip angle), and postcontrast 3D gradient recalled T1-weighted (34/3 ms; 1/0 mm) imaging.

Processing of DSC and DWI data was performed using a GE Advantage workstation running Functool software v4.4 (GE Medical Systems, Milwaukee, WI, USA). This allowed for the generation of cerebral blood volume (CBV), cerebral blood flow (CBF), and apparent diffusion coefficient (ADC) physiologic maps. The presence of reduced diffusion was defined as lesion-wide T2 ADC minimum <480 × 10^−6^ mm^2^/s (rADC_min_ 0.72) or ADC mean <1003 (rADC_mean_ 1.23), which is based on our prior experience in the diagnosis of nonenhancing glioma [20,21,22]. FMISO PET and MR sequences were co-registered using investigational GE PET/MR Review v1.0 software (GE Medical Systems, Milwaukee, WI, USA) allowing for the production of regions of interest (ROIs) and generation of quantitative values. DSC and DWI maps were standardized to contralateral normal appearing white matter (NAWM) allowing for the production of relative values. Semiquantitative tumor FMISO values were produced by standardization to ROIs placed on the left cerebellum (tumor to background; T/B value). T/B values above 1.2 were used to determine the hypoxic volume (HV) for each patient. Pearson product-moment correlation coefficient was utilized to measure for linear dependence between changes in hypoxic volume and perfusion metrics. *p* < 0.05 was considered statistically significant.

## 3. Results and Discussion

Pre-bevacizumab FMISO PET/MR imaging demonstrated a mass-like contrast enhancing lesion (mean ± standard deviation; 17.3 ± 19.7 mL) with surrounding T2/FLAIR signal hyperintensity (85.7 ± 61.0 mL) in all patients. Follow-up FMISO PET/MR imaging concurrent with bevacizumab therapy demonstrated markedly decreased contrast enhancing volume (1.39 ± 1.04 mL) with overall unchanged T2/FLAIR signal hyperintensity (90.3 ± 95.3 mL). The cohort baseline FMISO T/B_mean_ and T/B_max_ within the enhancing (1.13 ± 0.22 and 1.53 ± 0.55) and FLAIR hyperintense (0.95 ± 0.09 and 1.53 ± 055) component of the lesion was elevated compared to the follow-up examinations (enhancement, 1.12 ± 0.34 and 1.21 ± 0.46; FLAIR, 0.88 ± 0.12 and 1.21 ± 0.46). A statistically significant correlation between the percent reduction in rCBV and HV was observed within the cohort (*R* = 0.97, *p* = 0.03). No such correlation was observed between rCBF and HV.

**Figure 1:**
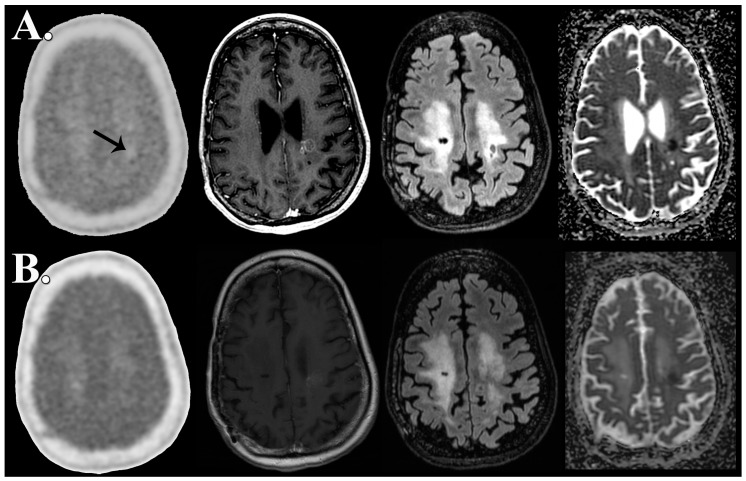
Abseent FMISO hypoxic volume in pseudoprogression prior to and following bevacizumab therapy. FMISO PET/MR imaging obtained simultaneously 7 days prior to (**A**) and 155 days after initiation of antiangiogenesis therapy (**B**) demonstrates pseudoprogression characterized by decreased volume of contrast enhancement (middle left) and nonenhancing FLAIR hyperintense mass (middle right) with associated focus of reduced diffusion (adc map, right). Unprocessed (left) and fused FMISO PET imaging (middle left, middle right, and right) demonstrates absence of FMISO accumulation above 1.2 time background. Pre-therapy FMISO PET/MR demonstrates slightly decreased focus of radiotracer uptake (upper left, arrow) relative to background which slightly increases to background uptake levels following bevacizumab therapy. FMISO HV is not observed at any time point in this patient with pseudoprogression.

**Figure 2:**
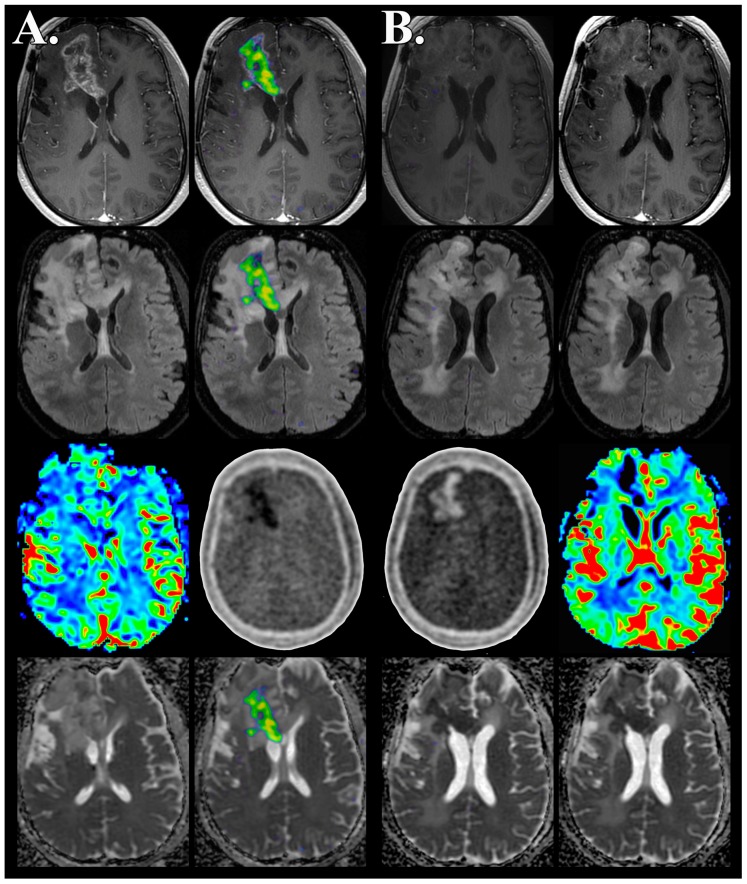
Absent FMISO hypoxic volume with associated development of reduced diffusion in recurrent high grade glioma concurrent with bevacizumab therapy. FMISO PET/MR imaging obtained simultaneously 5 days prior to (**A**) and 100 days following antiangiogenesis therapy (**B**) demonstrates progression of recurrent disease characterized by increased volume of nonenhancing mass (contrast, top row; FLAIR, top middle row) associated with the development of reduced diffusion (adc map, bottom row). Fused FMISO PET imaging (baseline, middle left; follow-up, middle right) demonstrates the resolution of tumor hypoxia concurrent with antiangiogenesis therapy. The unprocessed FMISO PET image (baseline, middle left; follow-up, middle right; bottom middle row) demonstrates the marked decrease in radiotracer accumulation below background tissue levels within regions of preserved cerebral blood volume (baseline, left; follow-up, right; bottom middle row) suggesting tissue consisting of highly cellular recurrent tumor with transformed or normalized tumor microvasculature.

Patient 4, who was ultimately diagnosed with pseudoprogression, demonstrated a persistent 1.06 mL focus of reduced diffusion on pre-therapy FMISO PET/MR imaging that was 0.86 mL at follow-up imaging (Figure 1). None of the patients ultimately diagnosed with recurrence demonstrated reduced diffusion at baseline examination, however, at follow-up, patient 2 and patient 3 both demonstrated a focus of non-enhancing reduced diffusion measuring 48.7 and 4.44 mL; respectively (Figure 2). The nonenhancing reduced diffusion lesions maintained CBV and CBF similar to normal appearing white matter.

**Figure 3:**
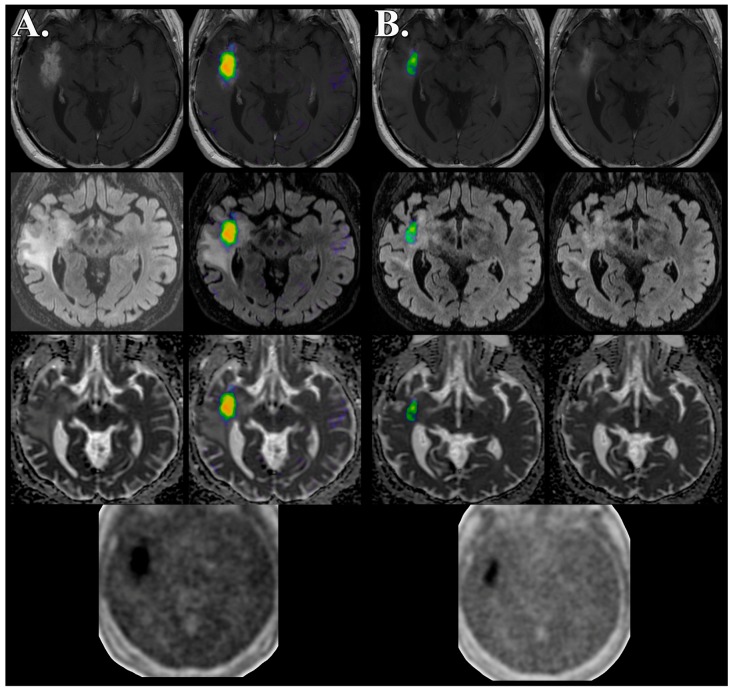
Decreased but persistent FMISO hypoxic volume in recurrent high grade glioma concurrent with bevacizumab therapy. FMISO PET/MR imaging obtained simultaneously 7 days prior to (**A**) and 36 days following antiangiogenesis therapy (**B**) demonstrates response to therapy characterized by decreased volume of contrast enhancement (top row) and nonenhancing FLAIR hyperintense mass (middle row) without evidence of reduced diffusion (adc map, bottom row). Unprocessed (bottom) and fused FMISO PET imaging (baseline, middle left; follow-up, middle right) demonstrates decreased but persistent tumor hypoxic volume predominately within the enhancing component. Follow-up MR imaging did not demonstrate the development of nonenhancing reduced diffusion mass at any point of therapy.

The three patients who were ultimately diagnosed with tumor recurrence demonstrated a baseline HV of 15.9 ± 18.7 mL that decreased to 2.28 ± 2.57 mL concurrent with bevacizumab therapy. The baseline HV T/B_mean_ and T/B_max_ in the recurrent disease group were 1.37 ± 0.14 and 1.72 ± 0.37, respectively. Concurrent with bevacizumab therapy, the recurrent disease group demonstrated a decreased HV T/B_mean_ and T/B_max_ of 1.30 ± 0.16 and 1.33 ± 0.47, respectively. Patient 1 demonstrated a decrease in the HV by 19.2% (Figure 3), however, this was markedly less than patients 2 and 3 who developed reduced diffusion within the nonenhancing lesion; 97.1% and 86.5%, respectively (Table 2 and Figure 2). In patient 2 and patient 3 the foci of reduced diffusion did not demonstrate a HV with T/B values greater than 1.2 (Figure 2).

Patient 4, who was ultimately diagnosed with pseudoprogression, demonstrated overall increased T/B values concurrent with bevacizumab therapy, however, did not demonstrate a significant HV at baseline or concurrent with bevacizumab therapy (Table 2). The patient with pseudoprogression was the only case of absent HV prior to bevacizumab therapy (Figure 1).

Our case series highlights the role molecular imaging can play as a tool for personalized diagnosis, prognosis, and monitoring of targeted therapeutics with the example of recurrent high grade glioma treated with antiangiogenic therapy. Three distinct features were observed from this small cohort of patients. First, the presence of pseudoprogression was retrospectively associated with the absence of hypoxia as demonstrated by FMISO PET/MR imaging. Second, a subgroup of patients with recurrent high grade glioma undergoing bevacizumab therapy demonstrated disease progression characterized by an enlarging nonenhancing mass with newly developed reduced diffusion, lack of hypoxia, and preserved CBV/CBF. Finally, a reduction in hypoxic volume was observed concurrent with targeted antiangiogenic therapy in all patients with recurrent tumor, however, markedly so in two patients that developed a nonenhancing reduced diffusion mass.

### 3.1. Utilization of Medical Imaging to Influence Personalized Medicine

Medical imaging has the potential to influence PM in several key aspects, especially involving molecular PET imaging for personalized diagnosis, patient specific disease prognosis, and therapeutic monitoring. Currently, data from large clinical trials guide most treatment decisions, often using a one-size-fits-all model and non-specific imaging endpoints. In the future, PM clinical decisions will increasingly be made based on patient-specific biological characteristics that will be directly evaluated by medical imaging. The use of targeted molecular PET imaging probes will allow for more precise disease monitoring throughout the course of therapy, allowing for early assessment of therapeutic response and detection of treatment failure. PET molecular imaging may allow for the delivery of patient specific therapy based on tumor imaging profiles and ability to noninvasively detect tumor response.

In the imminent future of PM, targeted molecular imaging will measure specific biological processes occurring before and concurrent with personalized therapy. Changes in these biological processes should be more sensitive measures of treatment response than morphological features such as RANO criteria. If a personalized therapeutic regiment is effective, as determined by direct monitoring of the targeted biological process, the therapy will be continued with high clinical confidence in the eventual outcome. Conversely, failure of a personalized therapeutic regiment to inhibit the targeted biological process will be directly detected early, affording the patient the benefit of alternative treatments.

Comprehensive integration of molecular imaging into PM requires close attention to designing clinical trials and selecting appropriate imaging biomarkers and a prognostic model that can serve as quantitative endpoints of the desired treatment outcome. This approach necessitates parallel development of both the test and the treatment concurrently. Such a model may move the medical community closer to the delivery of PM and achievement of real improvement in the clinical outcome.

### 3.2. The Role of Molecular Imaging in Personalized Medicine: Diagnosis

The observation from our case series that a patient with high grade glioma pseudoprogression demonstrated lack of hypoxia within the enhancing lesion highlights the potential role of molecular imaging as a tool in personalized diagnosis. The differentiation of glioma recurrence from pseudoprogression following the standardized treatment of maximal safe resection, radiation, and chemotherapy is critically important as the prognosis and treatment are completely different. The presence of increased contrast enhancement over time by MR imaging is nonspecific as it can be observed in the setting of pseudoprogression or the regrowth of tumor. The overlap of MR imaging appearance in these two disease processes presents a significant clinical dilemma when an enhancing lesion enlarges over time following the course of standard glioma therapy. However, on a biological level the difference between the two disease processes is marked. Pseudoprogression is likely the result of an inflammatory response to therapy resulting in permeability changes of the neurovascular unit [23,24,25]. Histologically, pseudoprogression is found to correspond to astrogliosis, inflammatory cells, and reactive radiation-induced changes without evidence of viable tumor [23,24,25,26].

In the traditional sense, information obtained from diagnostic neuroimaging primarily relies upon differences in brain morphology or signal changes induced by the compartmentalization of none-targeted exogenous contrast agents. This approach is often useful in demonstrating the extent of pathology within the brain, however, it is markedly limited in its ability to evaluate the diseases underlying cellular, molecular, or genetic pathways. As such the diagnostic and prognostic ability of standard imaging tools in differentiating recurrent tumor from pseudoprogression in patients with high grade glioma is markedly limited. The fundamental physiological differences in biology between the two entities provides an opportunity for targeted molecular imaging using hybrid PET/MR imaging that combines the molecular specificity of PET and the high soft tissue structural resolution of MR imaging.

PET imaging utilizes various radiotracers to noninvasively visualize specific biological processes and/or functional states other than hypoxia; for example, amino acid transport and metabolism and cell proliferation/membrane biosynthesis [27,28]. This unique imaging capability provides patient specific physiological information of biology within the glioma microenvironment that is not available from morphologic contrast enhanced MR imaging. The added value of PET molecular imaging has the potential to significantly advance the field of PM as it pertains to the care of glioma patients. ^18^F-Fluoroethyl-l-tyrosine (FET) and ^18^F-3,4-dihydroxy-6-(18)F-fluoro-l-phenylalanine (FDOPA) amino acid PET imaging have demonstrated the ability to differentiate disease recurrence from pseudoprogression in patients with high grade glioma [29,30,31,32,33]. Galldiks et al.’s investigation of 22 patients with suspected glioblastoma disease progression demonstrated that the pseudoprogression subgroup (*n* = 11) was characterized by reduced radiotracer uptake [30]. This study suggested that FET PET imaging may identify the subgroup prone to the development of pseudoprogression within the first 12 weeks following completion of chemoradiotherapy. Taken as a whole, these studies suggest that targeted PET imaging utilizing amino acid radiotracers is useful for diagnosing pseudoprogression with high sensitivity and specificity with the highest diagnostic utility obtained through a combination of static and dynamic FET PET metrics.

Hypoxia as a biomarker of glioma recurrence following standard of care therapy has yet to be fully investigated. The finding of lack of hypoxia within the contrast enhancing lesion in our patient ultimately diagnosed with pseudoprogression raises the possibility that static FMISO PET may serve act as a biomarker for PM diagnosis of pseudoprogression.

### 3.3. The Role of Molecular Imaging in Personalized Medicine: Prognosis

Aside from its diagnostic role, molecular imaging is likely to play an equally important role in predicting patient specific disease prognosis. The development of targeted molecular therapeutics will demand imaging capabilities beyond the typical roles of diagnostic screening, disease staging, and the generation of probability statements about disease etiology prior to tissue sampling. The appropriate molecular imaging contrast agent should be expected to demonstrate changes in the biological mechanism that was targeted by therapy. As such, the pre-treatment imaging would be expected to accurately select which patients will most likely benefit from a particular targeted therapy. In high grade glioma, the presence of hypoxia has been shown to be a prognostic factor in patient outcomes.

The use of FMISO PET as a targeted means of imaging chronic tissue hypoxia is not a new concept. The chronic hypoxic cellular microenvironment is a well-known trigger for a cascade of hypoxia inducible HIF-1a gene transcription responsible for cellular proliferation, migration, and angiogenesis resulting in increased therapeutic resistance [34,35,36,37]. Chronic hypoxia has been established to infer radioresistance, promote metastatic spread, and chemotherapy resistance for neoplastic cells [38,39]. FMISO was developed with the recognition that hypoxia plays an important role in many disease processes and there is a significant need for noninvasive in-vivo oxygen measurements [40,41,42,43,44,45].

The clinical FMISO PET procedure is relatively straight forward. A patient is administered approximately 3.7 MBq/kg of the radiotracer for conventional PET imaging. It should be recognized that time-of-flight PET detectors have the potential for significant dose reduction. Following an uptake time of 90 to 120 min the patient undergoes 20 to 40 min PET imaging with standard brain field of view. Image post processing and semiquantitative analysis is equally streamlined. On any standard imaging workstation, the amount of tissue that is hypoxic (HV) and the magnitude to which the tissue is hypoxic (T/B) can be determined. HV volume has been defined as the total number of pixels (excluding necrotic regions without uptake) with a T:B ≥ 1.2 [46]. HV does not require lesion demarcation and is unaffected by changes in cerebral perfusion once delivered to the brain parenchyma. The only requirement for tissue accumulation of FMISO is that the hypoxic cells are viable and that they exhibit active electron transport. The development of image-based blood surrogates for FMSIO quantification has shown that blood sampling is not necessary [16].

The extent and magnitude of tissue hypoxia in patients with glioma have been suggested as molecular imaging biomarkers that can contribute to personalized diagnosis and prognosis in patients with multiple disease processes, including glioma [47,48,49,50,51,52,53,54,55]. Swanson et al. has reported on 24 patients with glioblastoma who underwent contrast enhanced MRI and FMISO PET studies either prior to surgical intervention, after surgery but prior to radiation therapy, or after radiation therapy [48]. Abnormal regions observed by MR imaging were segmented, including the necrotic center, enhancing region, and nonenhancing T2 hyperintense region. The HV generally occupied a region straddling the enhancing and nonenhancing margin. Univariate survival analysis found HV to be the most significant predictor of survival. Spence et al. have also demonstrated a link between hypoxic tumor burden and clinical outcome in 22 patients with newly diagnosed glioblastoma [49]. FMISO PET imaging was used to calculate tumor HV and the maximum level of hypoxia in this cohort of patients prior to the initiation of combined chemoradiation therapy. Kaplan-Meier analysis demonstrated shorter progression free and overall survival in patients whose tumors contained elevated HV. Multivariate analyses for progression free and overall survival demonstrated that only FMISO measures of hypoxia were statically significant when comparing for MRI volume of enhancement, age, and Karnofsky performance status score.

The prognostic value of other PET radiotracers utilized for molecular imaging of glioma have also been reported. FET PET imaging has demonstrated the ability to predict clinical outcomes in patients with low grade glioma. Specifically, untreated glioma imaged with dynamic FET identified more aggressive tumors within the same WHO grade by quantification of reduced time-activity curves. Tumors with decreasing time-activity curves manifested earlier progression, higher rates of malignancy, and reduced overall survival [56,57,58,59].

### 3.4. The Role of Molecular Imaging in Personalized Medicine: Monitoring of Targeted Therapy

In neuro-oncology the traditional medical approach of “one size fits all” simply has not been successful in improving outcomes in patients with glioma. To this end, antiangiogenic therapies have been developed and tried on many patients who have failed initial temozolomide based chemotherapy. The rationale behind inhibiting angiogenesis as a therapy for glioma is robust. Angiogenesis is required for tumor growth beyond a millimeter owing to the limits of oxygen and nutrient diffusion within tissues [60]. As such, glioblastoma is highly vascularized and is often used as a preclinical tumor model of brain tumor angiogenesis [60,61,62]. The outgrowth of oxygen and nutrient supply by a tumor’s invasive margin results in hypoxia induced VEGF mediated angiogenesis [63,64,65,66]. Within newly diagnosed glioblastoma the highest levels of VEGF expression are observed within regions of cellular hypoxia and elevated microvascular hyperplasia [67].

Concurrent with the FDA approval of bevacizumab, multiple investigations were being undertaken to evaluate the effectiveness of targeted antiangiogenic therapy in patients with high-grade glioma. Many of these clinical trials used MR contrast enhancement as a primary clinical trial endpoint. Utilizing this criteria, nearly all of the studies demonstrated a reduction in lesion contrast enhancement and significantly improved response to therapy (progression free survival) [68,69,70]. The initial findings from these studies generated marked enthusiasm for the clinical role of targeted antiangiogenesis therapy. However, subsequent investigations failed to demonstrate an improved overall survival [71,72]. It should be noted that contrast enhancement is not a measure of active tumor tissue but a measure of neurovascular unity dysregulation through which gadolinium based contrast agent has leaked out.

The studies of targeted antiangiogenic therapy in recurrent glioblastoma have led to a reassessment of MR based diagnostic tumor response criteria [23]. Multiple reports have shown that targeted antiangiogenesis therapy of glioma results in reduced enhancing volume, likely due to normalization of the tumor microvasculature and reduced permeability of the neurovascular unit, however the lack of observed survival benefit highlights the need for more biologically relevant imaging biomarkers as a clinical trial endpoint in patients undergoing antiangiogenic therapy [73]. Molecular imaging may emerge as the method of choice for noninvasive monitoring of tumor response through the course of targeted therapy.

Multiple PET radiotracers have been investigated as potential imaging biomarkers to provide biological information about the response to targeted therapy that is more specific than contrast enhanced MR imaging. ^18^F-2-fluoro-2-deoxy-d-glucose (FDG) is commonly used for PET imaging of brain tumors but it has limited specificity. As a glucose analogue, it traces energy metabolism within the brain. Historically, FDG PET imaging of the brain suffers from high background signal, due to high baseline glucose metabolism, making identification of tumor signal challenging. As such FDG PET as a biomarker for antiangiogenesis therapy has not provided improvement over MR imaging. Kreisl et al. have reported on the use of FDG as an imaging biomarker in a Phase II clinical trial of bevacizumab in 31 recurrent anaplastic gliomas [74]. In this study, FDG uptake four weeks following the initiation of bevacizumab therapy was approximately 4% reduced with respect to pre-treatment imaging in 50% of the imaged cohort. This reduction from baseline was found to predict PFS, similar to MR contrast enhancement, however, was not predictive of OS.

Prospective studies have suggested that combining amino acid PET with contrast enhanced MR imaging may have more potential in assessing response to targeted antiangiogenesis therapy in patients with high grade glioma [75,76]. Hutterer and Galldiks et al. have each independently demonstrated that FET PET and MR imaging demonstrate discordant findings in up to 40% of cases in patients treated with bevacizumab [75,76]. Specifically, FET imaging analysis suggested that metabolic responders had overall improved survival when compared to nonresponders. FET PET was able to detect tumor progression earlier than MR imaging alone in cases of discordant PET and contrast enhancing MR imaging RANO criteria for response to therapy. Earlier detection of disease by FET PET provided up to 10 weeks of treatment benefit. Similarly, FDOPA PET imaging has promise in monitoring of response in patients with high grade glioma undergoing targeted antiangiogenesis therapy [77]. Schwarzenberg et al. imaged FDOPA tumor volume at multiple time points through the course of bevacizumab treatment; the 2 and 6 week points were shown to be the best predictors of PFS and OS. Response to therapy at six weeks was discordant in 33% of cases between FDOPA PET and RANO MR imaging characteristics [77]. As with FET PET, FDOPA PET imaging predicted treatment failure significantly earlier than contrast enhancing MR imaging resulting in an average a 7.2 week lead time benefit.

FMISO has also been investigated as a prognostic biomarker of chemoradiotherapy response in patients with glioma. Gerstner et al. has investigated if tumor vasculature and hypoxia, as measured with perfusion MRI and FMSIO PET, is prognostic of clinical outcomes in patients with newly diagnosed glioblastoma undergoing standard of care temozolimide base chemoradiotherapy [78]. In this prospective multicenter study it was determined that elevated tumor perfusion, vascular volume, and hypoxia is associated with reduced progression free and overall survival.

In our four patients with high grade glioma treated with bevacizumab, we observed three distinct patterns of FMISO PET/MR imaging characteristics. First, lack of hypoxia was a hallmark of pseudoprogression throughout the course of bevacizumab therapy. Second, 2 of the 3 patients with recurrent disease developed an enlarging nonenhancing mass associated with reduced diffusion, lack of hypoxia, and reduced CBV/CBF. Finally, all three patients with recurrent tumor demonstrated reduced hypoxic volume throughout the course of bevacizumab therapy; however, this reduction was more pronounced in the two patients with the nonenhancing reduced diffusion mass (97% and 87%) when compared to disease recurrence without reduced diffusion (19%). The small size of the cohort precludes an analysis of clinical outcome; however, our preliminary assessment suggests that serial FMISO PET imaging may play a role in the monitoring of patients undergoing bevacizumab therapy for progression of high grade glioma.

We believe the concurrent reduction of FMSIO HV in patients with recurrent glioma undergoing bevacizumab therapy is in part mediated by the biological interplay between tissue oxygenation and cerebral perfusion. Specifically, we hypothesize that bevacizumab induced vascular normalization results in improvement in tissue oxygenation. These findings are supported by histopathological evaluation of the effects of antiangiogenic therapies in patient-derived glioma specimens that demonstrate disappearance of microvascular proliferation, significant reduction of microvessel density, and reduction in endogenous molecular markers for tumor hypoxia suggesting improvement in tissue oxygenation [79]. These histological findings are supported by the observation in our cohort of a correlation between reduction in hypoxic volume and decreased rCBV occurring as a result of bevacizumab therapy. Additionally, prior studies have demonstrated that imaging biomarkers of glioma microvascular density and VEGF expression are reduced through the course of bevacizumab therapy [67,80]. Bevacizumab therapy results in a reduction in CBV and CBF metrics approaching baseline normal, appearing as white matter values, suggesting that a normalization of the tumor microvasculature can be captured noninvasively [81]. The mechanism by which a recurrent tumor characterized by reduced diffusion develops through the course of bevacizumab therapy remains to be established.

### 3.5. Advantages of Simultaneous PET/MR Imaging

Simultaneous PET/MR imaging provides unparalleled structural, metabolic, and functional information that contributes to its role as a tool for PM. Paramount to simultaneous imaging is patient convenience. PET/MR imaging provides a “one-stop shop” for medical imaging, rather than having to pass through several modalities, thereby decreasing total scan time and potential for repeated scanning. Additionally, simultaneous PET/MR imaging provides superior temporal resolution of imaging sequence acquisition. Logistically, it can be difficult for patients to obtain both clinical PET and MR imaging scans in a non-simultaneous manner within the same day. As such, clinical imaging sessions are often spread out over days if not weeks. This markedly limits the temporal resolution of non-simultaneous imaging data when attempting to understand the biological changes occurring through the course of therapy. The simultaneous acquisition of dynamic PET and MR imaging data negates the introduction of unnecessary variables by aligning temporal resolution of the imaging sequences. Finally, simultaneous PET/MR imaging with newer time of flight based digital detectors allows for marked dose reduction as high as 73% to the patient [82,83]. This degree of radiation dose reduction is incredibly important within the pediatric oncology population.

The utilization of simultaneous PET/MR imaging requires a method of attenuation correction not based on CT imaging. As such, MRI-guided attenuation correction (MRAC) methods rely on proton density and magnetic relaxation properties of tissues rather than electron density and photon attenuating properties of tissues, as would be the case for PET/CT imaging (CTAC). There is no unique mapping technique to convert MRI intensities to attenuation coefficients. However, derived attenuation maps can be utilized for segmentation of different tissue classes and assign proper linear attenuation coefficients to each tissue class. In brain PET imaging, the calvarium substantially contributes to the attenuation and scattering of annihilation photons [84]. For accurate PET quantification, the bones must, therefore, be accounted for within the MRAC map. Malone et al. previously compared, within the same dataset, attenuation correction utilizing MRAC approaches with the gold standard ^68^Ge techniques [84]. This group demonstrated that MRAC is an attractive option for attenuation correction of brain scans acquired on combined PET/MRI systems. Additionally, Spick et al. demonstrated that the clinical performance, in their cohort of 2300 patients, of PET/MRI and PET/CT is comparable [85]. Finally, multiple studies have demonstrated a high correlation of SUV values obtained utilizing MRAC compared to CTAC [82,83,84,85,86]. We therefore, do not believe that the methodology of utilizing MRAC as an attenuation correction methodology introduced confounding variables into the thresholding of FMSIO HV measurements in our study.

## 4. Conclusions

As highlighted by our case series, medical imaging has the potential to influence PM by contributing patient-specific tumor characteristics, precisely monitoring biologically specific markers of disease pathology through the course of therapy, and allowing for the development of patient-specific therapeutic regiments. Our initial experience suggests that the dynamic effects induced by bevacizumab therapy in patients with progressive high grade glioma can be captured by simultaneous FMISO PET/MR imaging. Specifically, the presence of tumor growth through the course of bevacizumab therapy is characterized by enlarging, nonenhancing, reduced diffusion mass that lacks hypoxia (FMISO accumulation). This constellation of imaging findings may serve as an earlier indicator of disease progression than contrast enhancement in a subgroup of patients undergoing bevacizumab therapy.

## Figures and Tables

**Table 1:** Patient demographics and clinical outcome.

Patient #	Age/Sex	Initial Dx	Molecular Profile	FMISO PET/MRI Time	Clinical Outcome
1	61/M	AA	IDH Wild	36	Recurrence
2	43/M	OA	IDH Mutant, 1P/19Q intact	100	Recurrence
3	65/M	AA	IDH Wild	46	Recurrence
4	52/W	AA	NOS	155	Pseudoprogression

Reference: M, man; W, woman; Dx, Diagnosis; AA, anaplastic astrocytoma; OA, oligoastrocytoma; IDH, isocitrate dehydrogenase; Wild, wild type without described gene mutation; Mutant, mutated gene detected; Intact, no evidence of gene deletion; NOS, not otherwise specified; FMISO PET/MRI Time, days elapsed time between baseline pre-therapy imaging and follow-up imaging.

**Table 2:** Percent change of 18F-fluoromisonidazole (FMISO) metrics between imaging time points.

Patient #	1	2	3	4	Mean	Standard Deviation
Hypoxic Volume	−19.2	−97.1	−86.5	0	−50.75	48.25
Hypoxic Volume T/B_mean_	−3.06	−18.2	5.78	NA	−5.16	10.23
Hypoxic Volume T/B_max_	−13.1	−31.1	−28.9	NA	−24.37	14.58
FLAIR Volume	−59.9	3.26	7.32	−14.1	−15.86	30.80
FLAIR T/B_mean_	2.51	−22.6	−1.09	14.1	−1.77	15.32
FLAIR T/B_max_	−27.9	−46.7	−51.4	62.1	−15.98	53.03
Enhancing Volume	−80.4	−96.9	−95.8	−41.4	−78.63	25.93
Enhancing T/B_mean_	34.1	−33.3	−1.92	11.5	2.60	28.17
Enhancing T/B_max_	−27.9	−46.7	−51.4	62.1	−15.98	53.03
Lesion rCBV	−22.0	−50.1	−46.2	−0.04	−30.5	17.3
Lesion rCBF	−18.4	−12.1	−0.04	−0.01	−0.22	0.08

Reference: NA, not applicable; T/B, tissue to background FMISO uptake ratio; max, maximum. Negative value represents decrease in percent change from baseline. Lesion rCBV and rCBF calculated at baseline (enhancing mass) and follow-up exam (reduced diffusion mass when present, or T2 hyperintense lesion when no reduced diffusion mass was present).
